# Psychological Functioning of Women Diagnosed with Lichen Planus and Other Diseases of the Oral Cavity—Explorative Study

**DOI:** 10.3390/healthcare11081118

**Published:** 2023-04-13

**Authors:** Urszula Sajewicz-Radtke, Bartosz M. Radtke, Paweł Jurek, Michał Olech, Anna Skurska, Zuzanna Ślebioda, Barbara Dorocka-Bobkowska, Katarzyna Pietuch, Magdalena Sulewska, Magdalena Błażek

**Affiliations:** 1 Laboratory of Psychological and Pedagogical Tests, Czarnieckiego 5A, 80-239 Gdansk, Poland; 2 Institute of Psychology, University of Gdansk, Bażyńskiego 4, 80-952 Gdansk, Poland; 3 Department of Psychology, Medical University of Gdańsk, M. Skłodowskiej-Curie 3a, 80-210 Gdansk, Poland; 4 Periodontologia Anna Skurska Private Dental Practice, Warszawska 14 lok. 2D, 15-063 Bialystok, Poland; 5 Department of Oral Surgery, Periodontology and Oral Mucosa Diseases, Poznan University of Medical Sciences, ul. Bukowska 70, 60-812 Poznan, Poland; 6 Department of Prosthetic Dentistry and Gerodontology, Poznan University of Medical Sciences, ul. Bukowska 70, 60-812 Poznan, Poland; 7 Department of Obstetrics, Gynecology and Oncological Gynecology, Medical University of Warsaw, Kondratowicza 8, 03-242 Warsaw, Poland; 8 PerioClinica Magdalena Sulewska Dental Practice, Kujawska 53/1, 15-548 Bialystok, Poland

**Keywords:** lichen planus, burning mouth syndrome, recurrent aphthous stomatitis, autoimmune, temperament, personality, self-esteem, social competences and functioning

## Abstract

The role of psychosocial factors in the development of changes in lichen planus and other diseases of the oral cavity has been implicated, but is still understudied. Therefore, the aim of our study was to describe the specific profile of psychological functioning of patients with these diseases, including the role of temperamental traits, action-oriented personality components, and self-esteem. In total, 94 adult women participated in the study: (1) with lichen planus (LP; *n* = 46; *M*_age_ = 54.80, *SD* = 12.53), (2) with other oral conditions (*n* = 25; *M_age_* = 34.76, *SD* = 16.03), (3) without chronic disease (*n* = 24; *M_age_* = 40.96, *SD* = 13.33). The following questionnaires were used: ZKA-PQ/SF, Polish Adaptive and Maladaptive Perfectionism Questionnaire, ACS-90, PROCOS, and MSEI. Results indicated no significant differences in temperament dimensions between studied groups. However, women diagnosed with LP presented lower levels of maladaptive perfectionism and social support than healthy women. Furthermore, women with LP also obtained lower scores for social resourcefulness and higher scores for moral self-approval than healthy women. Summarizing, patients with LP often use compensatory mechanisms that negatively affect their social functioning; thus diagnostic/therapeutic programs directed towards those group should be holistic, including psychologists and psychiatrists who support patients’ psychological well-being.

## 1. Introduction

Lichen planus (LP) is a chronic, recurrent inflammatory disease of unspecified etiology that affects the skin, mucosa, or both [1,2]. It is estimated that LP affects approximately 2% to 5% of the population, with a predominance in women (2:1) [3,4]. It is characterized by the appearance of white lesions with a reticular or lamellar pattern. Clinically, LP has been classified and divided into three subtypes: reticular, atrophic (erythematous), and erosive (ulcerative) [5,6]. According to the literature, lesions due to lichen planus are considered as a potentially malignant oral disorder with a risk of malignancy at a level of up to 12.5% [2,7,8,9,10].

LP was first described by Sir William James Erasmus Wilson in 1869, who characterized patients as restless, nervous, and vulnerable, with a tendency to worry excessively. Wilson also described patients as people exposed to periods of excessive emotional stress [11]. To this day, stress is considered to be one of the most common causes of disease exacerbations [7]. Historically, the occurrence of lichen planus has been associated with infection of the nervous system [12]. Researchers have also observed a relationship between the appearance of changes in LP and hormonal disorders that occur in the perimenopausal period in women, as well as diets low in retinol and beta carotene [13,14]. Nowadays, lichen planus is considered to belong to a large group of autoimmune diseases, the development of which depends on the presence of specific gene polymorphisms and point mutations. A number of factors related to impairment of the immune system and dysbiosis in the microflora of the oral cavity and intestines are responsible for the progression of changes [12]. The literature also shows a relationship between lichen planus and squamous cell carcinoma of the oral cavity [15]. Over many years various therapeutics have been used, but a consensus treatment guideline is still lacking [16]. Topical steroids and calcineurin inhibitors are most often used in the treatment of lichen planus. In the literature there are also reports on the use of low-level laser, CO_2_, and photodynamic therapy. In the case of very advanced symptomatic lesions, systemic therapy with corticosteroids or hydroxychloroquine are also recommended [16]. Due to the unspecified etiology of the disease, treatment of lichen planus is difficult and carries a high risk of failure, and many commonly-used drugs may worsen the situation [16]. Therapy should be multifaceted, focusing on optimizing the microbiome and restoring the proper functioning of the immune system by, inter alia, supporting processes to improve the patient’s mental state.

Burning mouth syndrome (BMS) is often characterized by a burning sensation, pain, or abnormal sensation in the oral mucosa, without visible pathological changes or known dental or medical causes [17]. Furthermore, clinical experience shows that discomfort may occur together with xerostomia, hypersensitivity to certain nutrients, and dysgeusia. Comorbid conditions include psychological conditions such as anxiety and depression, as well as nutritional deficiencies [18]. The etiopathogenesis of the disease remains unclear. Potential local factors contributing to the occurrence of BMS include mechanical irritation parafunctionalities, contact allergies to dental materials, dysfunctions of the stomatognathic system, as well as the phenomenon of electro-galvanic currents [17,19,20]. Systemic factors associated with the risk of BMS include diabetes mellitus, deficiencies of B vitamins (B1, B2, B6, and B12), deficiencies of folic acid and iron, hormonal disorders, gastrointestinal diseases, psychiatric and neurological disorders, and drug side effects [19,21]. Some published evidence hints at a link to trigeminal neuropathy, peripheral small fiber neuropathy, and centrally mediated pain. The latter can be related to dysfunctional dopaminergic neurons in the basal ganglia and may result in allodynia, dysesthesia, or hyperalgesia [22]. BMS is most often found in perimenopausal women [17]. Similar to LP, the unclear background of the disease leads to mainly symptomatic and often ineffective treatment, which may decrease the patient’s wellbeing.

Chronic recurrent aphthous stomatitis (RAS, recurrent aphthous ulcers, canker sores) is a chronic, ulcerative inflammatory disease of the oral mucosa; the reported frequency of which ranges from 5% to 20% [21,23]. Studies show this disease is more prevalent among white populations, women, non-smokers, and people with low socioeconomic status [24,25,26,27]. The etiopathogenesis of the disease is still not fully understood; however, it is assumed to be multifactorial [23]. Aphthous ulcers can appear alone or can be associated with some underlying disease [28]. The literature describes a relationship between aphthous ulcers and systemic diseases, such as mouth and genital ulcers with inflamed cartilage (MAGIC) syndrome or Crohn’s disease, and the use of medication such as non-steroidal anti-inflammatory drugs [29]. As in the case of LP and BMS, treatment is mainly symptomatic. In people with RAS, an inadequate response of the immune system to specific causative stimuli is observed; stimuli include mechanical injuries, stress, and bacterial and viral antigens [23,30]. However, the frequent occurrence of the disease among relatives indicates the possibility of genetic determinants. The inheritance of specific gene polymorphisms, especially those related to genes encoding pro-inflammatory cytokines involved in the formation of aphthous ulceration, may increase the predisposition to disease in members of a given family [31,32]. The genetic background of the disease and the mechanisms of impaired immune response have not yet been identified. In the course of RAS, painful single or multiple erosions and ulcers on the oral mucosa recur, which negatively affect the quality of life of patients.

In line with the biopsychosocial model, a growing body of evidence is increasingly implicating psychosocial factors in the development of changes in lichen planus, burning mouth syndrome, and chronic recurrent aphthous stomatitis; however, there is still little known about the specific psychological functioning of patients diagnosed with these diseases [19,21,33,34,35,36]. Studies indicate that patients with LP show increased levels of psychosocial stress [36] and suffer more often from anxiety and depression, which are the most common causes of change in oral tissues [34,37]. It is also observed that patients with diseases of the oral cavity report subjective increases of symptoms during times of higher life stressors [38]. Furthermore, these patients are less effective at coping and have experienced generally more stressful life events [11]. In addition, temperamental traits, as a biological aspect of formal behavioral characteristics, have been shown to be associated with diseases of the oral cavity [39]. Personality traits are correlated with health-related behaviors, such as coping with stressful situations, reporting illness symptoms, reactive action, and seeking social support [40]. Neuroticism is associated with experiencing more negative emotions as well as being more self-conscious, whereas extraversion is correlated with being more outgoing, open to other people, and assertive; thus, highly extraverted people are more likely to react to stress better and in more adaptive ways [39]. Previous research shows that patients with LP are highly self-controlled, conservative, and very norm-conscious [41]. Other research indicates that patients with LP tend to express their anger internally, thus, generating more tension, and when it is necessary, they express negative emotions in a non-adaptive way [42]. Patients with LP also experience problems with self-esteem and present a lower quality of life [43,44].

Therefore, based on the above theoretical background, the aim of this study was to describe the psychological functioning profile of a patient diagnosed with LP and other diseases of the oral cavity. For this study we created three explorative research questions:

1. Do temperamental traits as a biological aspect of formal behavioral characteristics differentiate healthy women from women suffering from LP and other diseases of the oral cavity?
2. Do action-oriented personality components differentiate healthy women from women suffering from LP and other diseases of the oral cavity?
3. Do women with lichen planus and other diseases of the oral cavity and healthy women differ in terms of different aspects of self-esteem?

## 2. Materials and Methods

### 2.1. Participants and Procedure

This study was designed as a multi-center case series study. The project was compliant with the Helsinki Declaration of 1975, as revised in 2000, and reviewed and approved by the Ethical Committee of Medical University of Gdansk (decision no. NKBBN/543/2020). Participants were recruited personally by doctors through one-on-one discussions from the Department of Medical University of Poznan, Mazowiecki Hospital in Warsaw and in a private practice in Bialystok, Poland, between the years 2020 and 2022. Written consent to participate in the study was collected from all participants. They were informed that participation in this research is completely voluntary and they could withdraw at any time; no payment for participation was provided.

A total of *N* = 94 adult women participated in the study, including *n* = 46 patients suffering from lichen planus, *n* = 25 patients with other oral conditions, and *n* = 24 women without any chronic disease. Table 1 presents detailed data on the characteristics of the surveyed women divided into the three comparison groups. As can be seen in Table 1, the groups differed in average age, which was due to the specificity of the experienced diseases and the fact that they all had similar treatment times. Inclusion criteria were as follows: In the case of erosive LP lesions—characteristic lesions and a confirmation through histopathological diagnosis; in the case of BMS—subjective experience of burning/numbness reported by the patient, no visible lesions on mucous membranes, and mycology and microbiology examination revealing only physiologically normal flora, and blood results showing no deficiencies in Fe, VitB12, or folic acid; and in the case of recurring mouth ulcers—the presence of characteristic lesions and history of occurrence in family members confirmed through interview. After each patient signed the consent documents and the inclusion criteria were verified, the patient received a set of paper–pencil questionnaires and was asked to fill them in honestly. There was no time limit.

### 2.2. Measures

To assess various aspects of the personality of the surveyed women (temperament, self-esteem, action-oriented traits), the following psychometric tools were used:

**The Zuckerman-Kuhlman-Aluja Personality Questionnaire Shortened Form (ZKA-PQ/SF)** [45]. This brief version of the questionnaire has 80 items (four items per facet) with a 4-point answer format (1—*strongly disagree*; 4—*strongly agree*) measuring five temperamental and biological main factors of personality: Aggressiveness, Activity, Extraversion, Neuroticism, and Impulsive-Sensation Seeking. Each of the main factors additionally consists of four facets of the personality, together giving a comprehensive multi-dimensional temperament profile of the respondent. The results of the validation study support the use of the ZKA-PQ/SF, given its good psychometric properties and strong equivalence to the long version (see [45]).

**The Polish Adaptive and Maladaptive Perfectionism Questionnaire** [46]. The questionnaire consists of 35 items with a 7-point answer format (1—*strongly disagree*; 7—*strongly agree*) measuring two dimensions of perfectionism: adaptive (e.g., “I persistently strive to achieve my goals”) and maladaptive (e.g., ”Whenever I make a mistake, I feel inferior to others”).

**Action Control Scale (ACS-90)** [47]. The ASC-90 consists of 36 items describing a particular situation with two alternative answers (A or B), one of which indicates an action orientation and the other a state orientation. The scale measures three variables: (1) failure-related action orientation vs. preoccupation; (2) decision-related action orientation vs. hesitation; and (3) performance-related action orientation vs. volatility.

**Multidimensional Questionnaire of Plans (MQP)** [48]. The MQP assesses the plans made and realized by an individual. The questionnaire contains 55 statements that are assessed on a 5-point Likert-type scale, where 1 indicates *full agreement* and 5 indicates *complete disagreement*. It consists of six subscales: (1) goal orientation—9 items (e.g., “I know what I want to achieve in life”); (2) planning—9 items (e.g., “I try to foresee all possibilities before starting to act”); (3) social support—11 items (e.g., “When I plan something, I think about what others will say about it”); (4) richness of life content—12 items (e.g., “I have a lot of goals in life that I try to realize”); (5) avoiding failures—7 items (e.g., “I fear difficult tasks”); (6) detailedness of planning—7 items (e.g., “I plan in detail what I will do in the future”).

**Profile of Social Competences (PROCOS)** [49]. This measures social competences and assesses their levels in five specific areas: assertive competence, cooperative competence, sociability competence, social resourcefulness, and community. It thus allows the profiling of respondents. The tool consists of 60 diagnostic items and 30 buffer items. All the items are descriptions of activities or tasks, and the respondent has to assess how good they are at a given activity or task on a 4-point scale (1—*definitely bad*; 4—*definitely good*).

**The Multidimensional Self-Esteem Inventory (MSEI)** [50,51]. This questionnaire measures self-esteem. It consists of 11 scales concerning self-esteem, its overall level, as well as eight components referring to particular aspects of a person’s functioning, such as competence, lovability, likability, personal power, self-control, moral self-approval, body appearance, and body functioning. Moreover, it contains a scale for assessment of the integrity of one’s self-concept, which reflects the effectiveness of the self-discovery process, and a scale allowing for the assessment of one’s need for social approval. The questionnaire consists of 116 test items assessed by the respondent on a 5-point scale.

## 3. Results

A one-way ANOVA was performed to compare the effect of the type of disease (lichen planus vs. no disease vs. other oral conditions) on various aspects of the women’s psychological functioning. Then, Bonferroni tests for multiple comparisons were used to identify significant differences in the studied variables between the group of women suffering from lichen planus and the other two groups of female respondents. The results of the analysis are presented in Table 2, Table 3 and Table 4, showing multidimensional temperament, action-oriented personality traits, and multidimensional self-esteem.

As can be seen in Table 2, there were no statistically significant differences between women suffering from lichen planus and the other two groups in terms of the examined dimensions of temperament.

A one-way ANOVA revealed that there were statistically significant differences between at least two groups in maladaptive perfectionism [*F(2, 92)* = 7.08, *p* < 0.01], social support [*F(2, 92)* = 5.10, *p* < 0.01], and avoiding failures [*F(2, 92)* = 6.44, *p* < 0.01]. The Bonferroni test for multiple comparisons found that women suffering from lichen planus scored lower for maladaptive perfectionism and social support than women with no disease (for details see Table 3).

Finally, as can be seen in Table 4, there were statistically significant differences between at least two groups in cooperative competence [*F(2, 92)* = 5.13, *p* < 0.01], social resourcefulness [*F(2, 92)* = 5.72, *p* < 0.01], moral self-approval [*F(2, 92)* = 5.00, *p* < 0.01], and defensive self-enhancement [*F(2, 92*) = 17.17, *p* < 0.01]. However, tests for multiple comparisons found that women suffering from lichen planus differed significantly from the other groups only in terms of social resourcefulness (women with no disease scored higher) and moral self-approval (women with no disease scored lower).

## 4. Discussion

The observed results suggest that participants who had diseases of the oral cavity also had poorer social functioning. The character of the condition causes difficulties in initiating and maintaining close relationships. The ailments experienced by such patients cause, at least sometimes, difficulties expressed as decreased levels of perceived social support. This obviously motivates the question of whether this is caused by actual difficulties or by avoidance of social contact due to worsening of symptoms and associated decreased self-esteem. These problems can manifest as the compensatory mechanism of decreased maladaptive perfectionism and a higher level of moral self-acceptance, which allows one to keep a positive self-image despite difficulties.

Our results present an interesting perspective in the ongoing discussion in the field. Lopez-Jornet and Camacho-Alonso [52] indicate that oral lichen planus (OLP) significantly decreases the quality of life of patients because of its strong influence on their social relations, psychological functioning, and everyday activity. Similar results were obtained by Alves et al. [53], who observed anxiety and depression in patients with OLP, as well as a negative influence of this condition on quality of life by affecting physical, psychological, and social spheres. Taking personality features of patients into account in some studies has provided interesting results [54]. In a study by Mohamadi Hasel et al. [39], it was found that factors associated with control, as well as neuroticism and extraversion, play an important role in predicting stress in patients. This suggests that personality dimensions can be risk factors or protective factors for patients faced with serious health problems, by influencing levels of experienced stress and depression and by causing relapse. The authors conclude that interventions aimed at reinforcing individual protective factors can be beneficial for decreasing patients’ stress and depression [39]. In another study, a significant relationship was observed between two personality features—diligence and extraversion—and the perceived improvement of OLP. Those with higher diligence had higher perceived quality of life in the context of oral health. Extraversion had a significant influence on decreased severity of clinical symptoms [54]. The results of our study indicate that social resources and the ability to cooperate have an important role and they are decreased as a result of the disease. Taking into account the results of other studies, one could conclude that this sphere is particularly challenging for patients, as their social functioning is negatively impacted and, as a consequence, their levels of social competence decrease. This issue definitely requires both further research and targeted interventions.

The influence of mucosal lesions, related side-effects, long and sometimes ineffective therapies, and the high recurrence rate can together greatly impair the patients’ physical health, emotions and social relations. The problem of psychological functioning is also discussed in other diseases characterized as autoimmune, such as Pemphigus Vulgaris (PV). Calabria et al. [55] reported high prevalence of psychiatric distress in their sample of PV patients. Anxiety and depression were found in 60.0% and 50.0% of the subjects, especially in females, who experienced higher levels of mood disorders than males. However, Pemphigus Vulgaris is a rare autoimmune muco-cutaneous disease. In contrast, diseases we selected for comparison are the leading, most common diseases in medical practice. Patients with lichen planus are the majority of patients with mucosal problems in dental office.

As in the study conducted by Vilar-Villanuev et al. [56], the results of our study highlight the need to provide OLP patients psychological help, especially in the areas of self-esteem and improving social relations. Psychological problems play an important role as a risk factor for the occurrence of OLP and relapses. Psychological help should be recommended in order to improve patients’ mental health [57], which would have a positive impact on their quality of life [58,59] and would lead to better disease treatment results [54].

## 5. Conclusions

The main goal of the current research was to describe the profile of psychological functioning of patients diagnosed with LP and other diseases of the oral cavity. Our results confirmed that psychosocial factors may play an important role in the quality of life and pathological changes presented by participants with LP and other diseases of the oral cavity, such as burning mouth syndrome and chronic recurrent aphthous stomatitis. Our results suggested that the above groups of patients did not differ significantly in terms of examined temperament dimensions; however, differences were found in action-oriented personality components—women suffering from LP had lower results for maladaptive perfectionism and social support than did healthy women. Furthermore, since previous studies have shown the importance of self-esteem in oral cavity diseases, we investigated whether the study groups differed in this regard. Differences were observed only for two aspects of self-esteem. Women with LP obtained lower scores for social resourcefulness and higher scores for moral self-approval compared to healthy women. Based on the obtained results, it can be concluded that women with diseases of the oral cavity use compensatory mechanisms that negatively affect their social functioning, mainly manifested in difficulties establishing and maintaining close relationships. Therefore, it is important that in the case of patients with the above diseases, diagnosis and therapy programs should be holistic and include specialists who can also support the patient’s mental state, such as psychologists and psychiatrists. It would also be helpful to create support groups to encourage these women to participate in society.

The above results indicate that the profile of psychological functioning of patients diagnosed with LP and other diseases of the oral cavity is very complex, heterogeneous, and ambiguous. We have identified some of the areas that need special support in these patients, but undoubtedly this requires further research and exploration.

## Figures and Tables

**Table 1:** Sample composition.

Variable	Lichen Planus (*n* = 46)	No Conditions (*n* = 24)	Other Oral Conditions (*n* = 25)
Age	*M* = 54.80, *SD* = 12.53	*M* = 40.96, *SD* = 13.33	*M* = 34.76, *SD* = 16.03
Time since onset of the condition (years)	*M* = 5.11, *SD* = 7.83	–	*M* = 7.70, *SD* = 9.44
Duration of treatment (years)	*M* = 4.73, *SD* = 7.76	–	*M* = 7.90, *SD* = 9.68
Concomitant diseases			
*Diabetes*	13.0%	–	0%
*Cardiovascular diseases*	34.8%	–	8.0%
*Thyroid diseases*	26.1%	–	16.0%
*Gastrointestinal diseases*	10.9%	–	24.0%
*Respiratory diseases*	4.3%	–	8.0%
*Hematopoietic system diseases*	0%	–	0%
*Skin diseases*	15.2%	–	4.0%
*Allergy*	8.7%	–	32.0%
*Rheumatoid conditions*	10.9%	–	0%
*Cancers/tumors*	4.3%	–	0%
*Mental disorders*	4.3%	–	8.0%
Medication taken			
*Psychotropic medication*	2.2%	–	8.0%
*Anti-diabetic medication*	13.0%	–	0%
*Cardiovascular medication*	30.4%	–	8.0%
*Gastrointestinal medication*	10.9%	–	12.0%
*Analgesics*	13.0%	–	8.0%
*Hormonal treatment*	13.0%	–	16.0%
*Steroid medication*	4.3%	–	0%
*Antibiotics*	0%	–	4.0%
*Other medications*	30.4%	–	8.0%
Frequency of doctor visits			
*4*–*6 times a month*	6.5%	–	4.0%
*2*–*3 times a month*	2.2%	–	0%
*Once a month*	4.3%	–	4.0%
*Once every two months*	6.5%	–	4.0%
*Once every 3–4 months*	19.6%	–	0%
*Once every 5–6 months*	34.8%	–	4.0%
*Less than once every 6 months*	26.1%	–	84%
The burdensomeness of the primary disease			
The patient reports no symptoms.	17.4%		28.0%
The symptoms of the disease moderately impair everyday functioning	65.2%		52.0%
The symptoms of the disease significantly impair everyday functioning	17.4%		20.0%
Substance use			
Smoking cigarettes	17.4%	12.5%	16.0%
Regular alcohol consumption	6.5%	4.2%	28.0%
Work and education			
*Employed*	47.8%	79.2%	44.0%
*Currently not employed*	8.7%	4.2%	4.0%
*Studying*	0%	4.2%	40.0%
*Retired/pension*	39.1%	12.5%	12.0%
*Retired/pension and working*	4.3%	0%	0%

**Table 2:** Comparison of the mean results of patients with lichen planus with results of other respondents—dimensions of temperament.

Variable	Lichen Planus (*M*)	Lichen Planus (*SD*)	Difference in the Means Compared to the Group of Lichen Planus Patients (Post Hoc Tests)		ANOVA— *F* Statistic
			No Diseases	Other Mouth Diseases	
Physical Aggression	6.50	2.23	0.67	−0.62	1.73
Verbal Aggression	9.67	2.44	−0.70	−1.53	2.59
Anger	8.76	2.58	0.39	−0.84	1.27
Hostility	8.67	1.99	0.13	−0.81	1.23
Work Compulsion	8.52	2.71	0.40	0.52	1.52
General Activity	11.18	2.35	−0.03	0.78	0.40
Restlessness	9.43	2.09	0.35	0.31	0.83
Work Energy	12.15	2.42	−0.56	0.67	1.76
Positive Emotions	11.41	1.75	−0.84	−0.43	1.51
Social Warmth	11.26	2.49	0.17	−0.82	0.93
Exhibitionism	9.74	2.59	−0.35	−1.22	1.82
Sociability	11.02	1.91	−0.73	−0.86	1.65
Anxiety	10.00	2.84	1.09	−0.36	1.51
Depression	10.22	2.40	1.26	−0.06	2.04
Dependency	10.59	2.11	0.96	0.23	1.16
Low Self-Esteem	9.91	2.41	1.74	0.99	3.57
Thrill and Adventure Seeking	8.09	3.13	0.75	−0.15	0.69
Experience Seeking	9.28	2.54	−1.51	−1.68	4.39
Disinhibition	8.02	2.59	0.36	−1.42	3.37
Boredom Susceptibility/Impulsivity	10.04	2.31	0.17	−0.56	0.82
Aggressiveness	33.61	7.15	0.48	−4.27	2.72
Activity	41.09	7.39	−0.04	2.09	0.79
Extraversion	43.43	5.78	−1.38	−3.33	1.91
Neuroticism	40.72	7.65	4.22	0.80	1.66
Impulsive-Sensation Seeking	35.43	7.66	−0.23	−3.81	2.49

*Notes. N* = 94.

**Table 3:** Comparison of mean results of patients with lichen planus with results of other respondents—components of personality associated with action orientation.

Variable	Lichen Planus (*M*)	Lichen Planus (*SD*)	Difference in the Means Compared to the Group of Lichen Planus Patients (Post Hoc Tests)		ANOVA— *F* Statistic
			No Diseases	Other Mouth Diseases	
Adaptive perfectionism	4.97	0.93	0.10	0.07	0.11
Maladaptive perfectionism	3.62	1.00	0.91 **	0.28	7.08 **
Action orientation subsequent to failure	11.57	3.38	−0.35	0.77	0.75
Decision-related action orientation	7.11	3.04	−0.68	1.71	4.11
Action orientation in pleasant situations	8.11	2.23	−1.27	−0.41	2.78
Goal orientation	3.51	0.61	−0.09	−0.04	0.14
Planning	3.95	0.45	0.23	0.20	1.51
Social support	3.49	0.53	0.54 **	0.31	5.10 **
Richness of life content	3.68	0.48	−0.18	0.02	1.00
Avoiding failures	3.39	0.65	0.47	0.62 **	6.44 **
Detailedness	3.38	0.55	0.30	0.31	2.14

*Notes.* ** *p* < 0.01.

**Table 4:** Comparison of mean results of patients with lichen planus with other respondents—multidimensional self-esteem.

Variable	Lichen Planus (*M*)	Lichen Planus (*SD*)	Difference in the Means Compared to the Group of Lichen Planus Patients (Post Hoc Tests)		ANOVA— *F* Statistic
			No Diseases	Other Mouth Diseases	
Assertive competence	34.78	5.87	3.24	3.70	3.49
Cooperative competence	33.78	6.84	5.07	3.86	5.13 **
Sociability competence	26.48	5.54	1.44	2.76	1.61
Social resourcefulness	28.02	5.96	5.06 **	2.14	5.72 **
Community competence	14.74	2.78	1.07	0.78	1.10
Competence	32.52	4.81	−3.64	−1.84	3.93
Lovability	36.17	7.91	−1.38	−0.46	0.38
Likability	33.65	5.53	−0.93	−1.31	0.51
Personal power	30.54	5.62	−0.71	−1.90	0.83
Self-control	26.50	4.77	−2.17	1.18	3.06
Moral self-approval	37.57	6.51	−4.68 **	−0.71	5.00 **
Body appearance	30.54	4.98	−2.37	−0.34	1.09
Body functioning	30.78	5.57	−2.30	−0.50	0.92
Identity integration	31.85	5.09	−1.90	0.81	1.42
Defensive self-enhancement	52.63	6.62	−3.37	7.35 **	17.17 **
Global self-esteem	31.57	5.08	−2.23	−0.43	0.98

*Notes.* ** *p* < 0.01.

## Data Availability

The data presented in this study are available on request from the corresponding author.
